# Violence against healthcare workers during coronavirus (COVID-19) pandemic in Egypt: a cross-sectional study

**DOI:** 10.1186/s41935-022-00304-3

**Published:** 2022-10-14

**Authors:** Noha M. Abu Bakr Elsaid, Omneya Ibrahim, Zeinab F. Abdel-Fatah, Hend A. Hassan, MennatAllah H. Hegazy, Marwa M. Anwar, Hanan H. Soliman

**Affiliations:** 1 Department of Public Health, Community, Environmental and Occupational Medicine, Faculty of Medicine, Suez Canal University, Ismailia, Egypt; 2 Department of Basic Medical Sciences, Faculty of Medicine, King Salman International University, South Sinai, Egypt; 3 Psychiatry & Neurology Department, Faculty of Medicine, Suez Canal University, Ismailia, Egypt; 4 Public Health and Community Medicine Department, Suez University, Suez, Egypt; 5 Theodor Bilharz Research Institute, Giza, Egypt; 6 Forensic Medicine & Clinical Toxicology Department, Faculty of Medicine, Suez Canal University, Ismailia, Egypt

**Keywords:** Healthcare workers, COVID-19 pandemic, Types of violence, Workplace violence, Egypt

## Abstract

**Background:**

Healthcare workers are on the front lines of COVID-19 and are subject to risks. A rise in the cases of violence and aggressiveness against HCWs has been observed worldwide, adding to the already existing burnout. The purpose of this research is to determine the prevalence of workplace violence, its risk variables, and the pattern of violence directed towards healthcare workers in the context of COVID-19 pandemic. The research used a cross-sectional analytic design. Purposive sampling was utilized to identify research participants using an online survey. Form’s link was distributed to accessible social media groups such as Facebook and WhatsApp from July 2020 to the end of October 2020. A self-administered structured survey was adapted from the World Health Organization survey questionnaire about violence in healthcare settings. The Google Form’s link was distributed to the social media groups until the total sample of 405 was collected.

**Results:**

During the COVID-19 pandemic, workplace violence against Egyptian healthcare workers was prevalent (63.2%). The most prevailing type of violence among the exposed participants was verbal violence (87.9%). Violence is more common in the (< 40 years old) age group (80.9% of exposed healthcare workers). Violence was more statistically significant against females (60.5% of the exposed healthcare workers) (*p*-value = 0.023). Regarding the work specialty, violence was more committed against physicians (84.3% of exposed healthcare workers) than nurses (12.8% of exposed healthcare workers). The primary perpetrators of violence were the patient’s family (74.6%). The majority of the exposed HCWs (96%) reported no physical injury from the violent event, and 71.5% deemed the violent incident preventable. The majority (90.6%) of HCWs exposed to violent incidents declared non-reporting.

**Conclusions:**

Effective risk communication at all levels of society is critical for reducing fear, stigma, and ultimately workplace violence, as recent assaults on healthcare institutions demonstrate. To reduce violence and safeguard the safety of the medical profession, the government, health policymakers, media organizations, and community engagement groups must collaborate for healthcare workers’ safety.

## Background

The epidemic of COVID-19 has sparked an uptick in violence against healthcare workers (HCWs). In response to numerous assaults and harassment of physicians and healthcare workers engaged in COVID-19 care or contact tracing, the Indian government approved a law-designating violence against healthcare employees as a non-bailable crime punishable by up to 7 years in prison (Vento, Cainelli, & Vallone, 2020)*.*

While HCWs have been praised as heroes in numerous nations for their actions during the COVID-19 outbreak, their efforts are not universally appreciated. Since the epidemic started, headlines have included instances of HCWs who have been subjected to violent attacks. Nurses and doctors have been physically assaulted in Mexico (Rodriguez-Bolanos et al., 2020). HCWs have been attacked, stoned, spat on, threatened, and expelled from their homes across India, according to reports. Unfortunately, violence against HCWs is not a new occurrence. Such attacks were becoming more common preceding the COVID-19 epidemic, in clinics and hospitals worldwide (McKay et al., 2020).

Workplace violence (WPV) is defined by the World Health Organization (WHO) as “incidents in which workers are mistreated, threatened, or attacked in the circumstances related to their job, presenting an explicit or implicit danger to their safety, well-being, or health.” The WHO’s definition of verbal and physical aggression was also used to express different forms of workplace violence. Physical violence included slapping, stabbing, pushing, biting, and pinching, while verbal violence included being screamed at, sworn at, humiliated, and threatened with danger and vulgar words (Di Martino, 2002).

Experiencing violence at work has several detrimental consequences on organizational and individual levels, including increased burnout perceptions, decreased job performance and satisfaction, poor mental health, an adverse work atmosphere, and less effective patient care (ALBashtawy & Aljezawi, 2016).

Before the COVID-19 pandemic, WPV prevalence varies by country. In Egypt, 69.5% and 9.3% of nurses, respectively, were subjected to verbal and physical assault (Abbas et al., 2010). Nurses were more subjected to WPV than physicians (*p* < 0.001) in Saudi hospitals, with over two-thirds (67.4%) reporting being vulnerable to violence (Algwaiz & Alghanim, 2012). A total of 75% of nurse workers in Jordan’s emergency services had been subjected to some sort of violence (ALBashtawy & Aljezawi, 2016). The majority of healthcare workers (80.4%) at Palestinian public hospitals reported being exposed to violence in the prior 12 months, with 20.8% reporting physical violence and 59.6% reporting nonphysical abuse (Hamdan & Hamra, 2015). In Turkey, 72.3% (141/195) of emergency personnel had been subjected to some violence (Boz et al., 2006).

During health emergencies, there are various reasons why people attack and mistreat healthcare providers. Fear, panic, misunderstanding regarding the spread of COVID-19, and misguided rage are possible drivers in various contexts during the COVID-19 pandemic. Only some government officials have replied by warning fast and harsh punishment for anybody who intentionally hurt healthcare personnel. On the other hand, threats of revenge do not address the underlying causes of such violence and are unlikely to be effective in ending it. Root causes must be addressed in order for effective responses (Pujadas et al., 2020).

This study aimed to safeguard HCWs by establishing a baseline of violence in light of the epidemic of COVID-19 so that measures to avoid and respond to violence against HCWs in Egypt could be developed. The objectives of the study included the following: (1) to determine the frequency of workplace violence against healthcare workers in Egypt during COVID-19, (2) to ascertain the forms of workplace violence perpetrated against HCWs in Egypt during COVID-19, (3) to determine risk variables for workplace violence against HCWs in Egypt during COVID-19, (4) to outline the frequency of health consequences of workplace violence directed against HCWs in Egypt during COVID-19, (5) to illustrate the opinions of HCWs regarding workplace characteristics that can prevent violence, and (6) to explore the reasons behind the occurrence of violence and the reasons of the non-reporting it. Research question is as follows: Was there an upsurge in the prevalence of workplace violence against HCWs during COVID-19 in Egypt? Research hypothesis is as follows:

- Alternative hypothesis (HA): There was an upsurge in the prevalence of workplace violence against HCWs during COVID-19 in Egypt.
- Null hypothesis (h0): There was no upsurge in the prevalence of workplace violence against HCWs during COVID-19 in Egypt.

## Methods

### Study design

The frequency of workplace violence against HCWs was determined using a cross-sectional survey in Egypt during COVID-19.

### Study setting

The study was conducted in Egypt, formally the Arab Republic of Egypt, a transcontinental republic that spans the northeast corner of Africa and the southwest corner of Asia through the Sinai Peninsula. Egypt is the Arab world’s most populous nation and the third most populous on the African continent, with over 98 million residents in 2019, distributed according to sex into 47,554 females and 50,547 males (Shechter, 2018).

### Study subjects

The sample size was calculated using one proportion sampling equation. The prevalence of the violence against HCWs in Egypt in 2017 was 59.7 (Abdellah & Salama, 2017), with a level of confidence of 95%, accuracy of 5%, and a design effect of 1, so the sampling size was 369. Then the sample size was upsized to 405 after applying a 10% (36) non-response rate. A purposive sampling technique was used to select study participants using an online survey. Form’s link was distributed to social media groups such as Facebook and WhatsApp applications from July 2020 until October 2020. Responses were collected until the completion of the required sample.

### Study participants’ criteria

All healthcare workers, including physicians, pharmacists, nurses caring for suspected or confirmed cases of COVID-19, aged ≥ 18 years, and who provided electronic informed consent, were included in this study, except those on annual leaves.

### Data collection tools

Due to the risk of contagion, traditional face-to-face interviews could not be adopted. Data was collected using an online questionnaire on Google Forms. Participants filled out a structured self-administered questionnaire adapted from a WHO survey on violence in healthcare settings (ILO & WHO, 2003).The respondents’ sociodemographic information, workplace properties, the prevalence of violent incidents in the preceding 3 months of the COVID-19 pandemic, risk factors contributing to workplace violence, personal perspectives, perceptions, attitudes, experiences, and suggestions about workplace violence were all included in the questionnaire. The questionnaire is valid, reliable, and available in Arabic (Abbas et al., 2010).

### Data management

The data was analyzed using the Statistical Package for Social Sciences (SPSS) software version 20.0 (Armonk, NY, USA: IBM Corp). The Kolmogorov–Smirnov test was performed to determine the normality of the analyzed variables’ distributions. The chi-square test (Fisher or Monte Carlo) was used to compare categorical variables, whilst the odds ratio (OR) was employed to quantify the ratio of the chances of an event happening in one risk group compared to the odds in the other non-risk group at a 95% confidence interval.

### Ethical considerations

The Suez Canal University’s Medical Ethics Committee approved the research on July 17, 2020, with approval number 4219. Informed consent was written at the top of the Google Form, and participants could choose whether or not to fill it out. The information gathered was kept private and confidential for the sole purpose of the research. The participants were informed that participation is completely optional, and that they may withdraw at any moment without providing an explanation.

## Results

The study involved 405 HCWs from different Egyptian governorates who enrolled and completed the electronic survey (Table 1). The vast majority (83.7%) of them was less than 40 years old, with a mean age of 33.73 ± 6.87 years. Female sex predominance was noted, representing 64.7% of the sample. Most of the participants were physicians (84.2%), followed by nurses (11.4%), and, lastly, pharmacists (3.7%). Most of the participants were involved in morning shifts.

**Table 1:** Distribution of the studied participants according to demographic characteristics (*n*** = **405)

Q	Demographic characteristics	No	%
**1**	**Age (years)**		
	< 40	339	83.7
	≥ 40	66	16.3
	Mean ± SD	33.73 ± 6.87	
	Median (min.–max.)	32.0 (21.0–70.0)	
**2**	**Gender**		
	Males	143	35.3
	Females	262	64.7
**3**	**Specialty**		
	Physician	341	84.2
	Nurse	46	11.4
	Pharmacist	15	3.7
	Others	3	0.7
**5**	**Work schedule**^**a**^		
	Morning	291	71.9
	Evening	184	45.4
	Night	8	2.0
	Changing	120	29.6

^a^ More than one answer

**Fig. 1:**
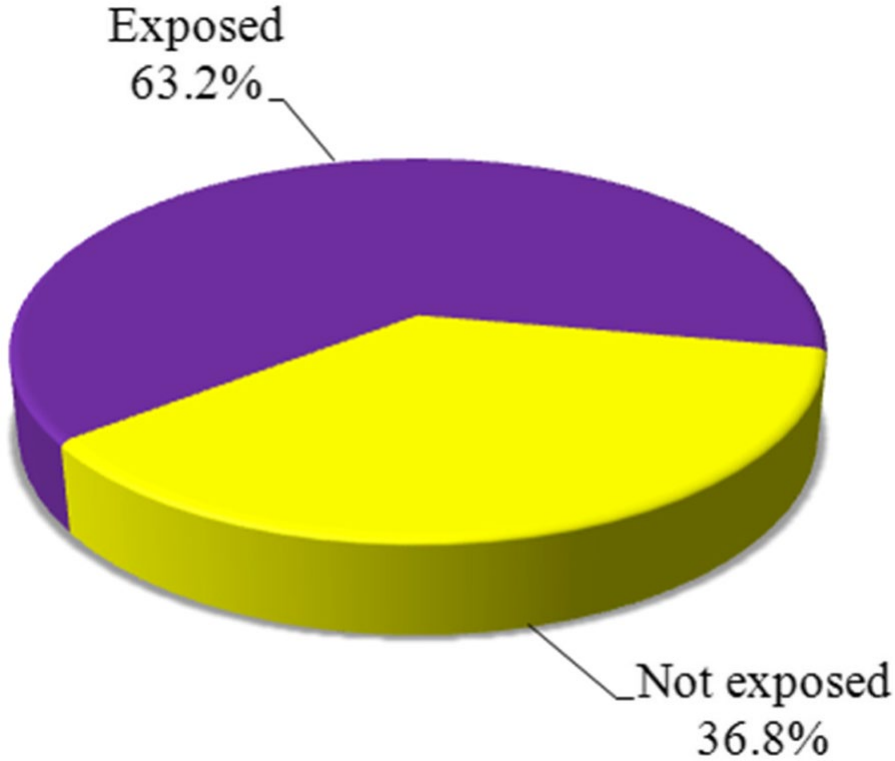
The prevalence of workplace violence exposure among the studied healthcare workers in context with the COVID-19 pandemic in Egypt (*n* = 405)

**Fig. 2:**
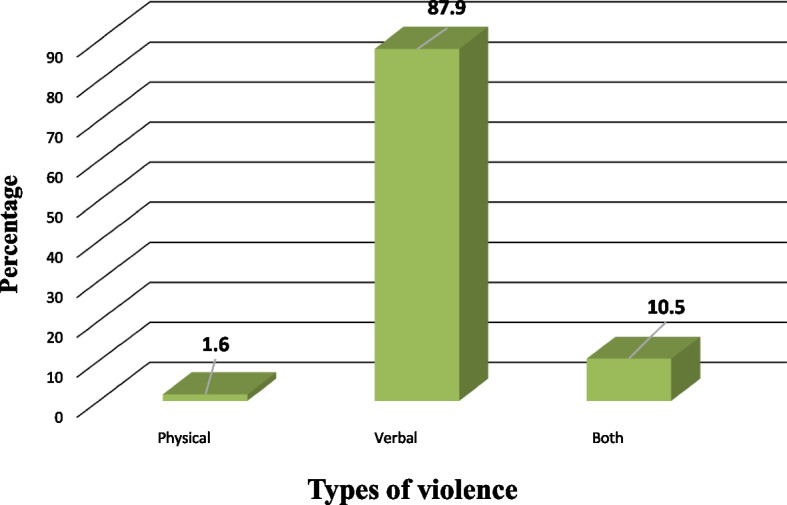
The pattern of workplace violence among the exposed healthcare workers in our study in the context of the COVID-19 pandemic in Egypt (*n* = 256)

Figure 1 shows that the prevalence of WPV against the studied Egyptian healthcare workers during the COVID-19 pandemic was 63.2% compared to 36.8% of nonexposed to violence. In Fig. 2, verbal violence was the most prevailing type of violence among the exposed HCWs (87.9%) compared to physical violence, which represents only 1.6%. It was noted that 10.5% of HCWs were exposed to both verbal and physical violence.

Table 2 illustrates the risk factors for WPV exposure among the studied HCWs. It was found that violence against the exposed HCWs is more common with statistically significant (*p*-value = 0.042) in the (< 40 years old) age group (80.9% of exposed HCWs) than the (> = 40 years old) age group (19.1% of the exposed HCWs). Violence was statistically significant against the female HCWs (60.5% of the exposed HCWs) (*p*-value = 0.023). Regarding the work specialty, violence was found to be much more committed against physicians (84.3% of exposed HCWs), followed by nurses (12.8% of exposed HCWs), and the least exposed were pharmacists (2.7% of exposed HCWs) with a *p*-value of 0.041. The violence happened to be more statistically significant during the evening shifts (*p*-value =  < 0.001). Violence also showed statistical significance where the number of colleagues in the same place of work equals (6–10) (*p*-value =  < 0.001).

**Table 2:** Risk factors of workplace violence among the studied healthcare workers in context with the COVID-19 pandemic in Egypt according to their demographic characteristics

Demographic characteristics	Exposure to violence					Total (*n* = 405)		*χ*^2^	*p*	OR (95% *CI*)	*p*
	**Exposed (*n* = 256)**			**Not exposed** **(*n* = 149)**							
	**No**	**%**		**No**	**%**	**No**	**%**				
**Age (years)**											
< 40	207	61.1		132	38.9	339	83.7	4.127*	0.042*	0.544 (0.301–0.985)	0.044*
≥ 40^b^	49	74.2		17	25.8	66	16.3			-	–
**Gender**											
Males	101	70.6		42	29.4	143	35.3	5.233*	0.022*	1.660 (1.073–2.568)	0.023*
Females^b^	155	59.2		107	40.8	262	64.7			–	–
**Specialty**											
Physician^b^	216	63.3		125	36.7	341	84.2	7.691*	0.041*		
Nurse	33	71.7		13	28.3	46	11.4			1.469 (0.745–2.895)	0.267
Pharmacist	7	46.7		8	53.3	15	3.7			0.506(0.179–1.430)	0.199
Others	0	0.0		3	100.0	3	0.7			0.0 (0.0)	0.999
**Work schedule**^**a**^											
Morning	182	62.5		109	37.5	291	71.9	0.198	0.657	0.903 (0.574–1.418)	0.657
Evening	141	76.6		43	23.4	184	45.4	26.116*	< 0.001*	3.022 (1.963–4.653)	< 0.001*
Night	6	75.0		2	25.0	8	2.0	0.488	^FE^*p* = 0.716	1.764 (0.351–8.853)	0.490
Changing	78	65.0		42	35.0	120	29.6	0.235	0.628	1.116 (0.715–1.742)	0.628
**Number of colleagues in the same place of work**											
None	16	69.6		7	30.4	23	5.7	12.992*	0.005*	0.997 (0.381–2.606)	0.995
1–5	109	65.3		58	34.7	167	41.2			0.820 (0.504–1.332)	0.423
6–10	37	46.3		43	53.8	80	19.8			0.375 (0.212–0.665)	0.001*
> 10^b^	94	69.6		41	30.4	135	33.3			–	–
**There is a system for reporting violence**											
Yes^b^	**152**	**71.0**		**62**	**29.0**	**214**	**52.8**	11.927*	0.001*	–	–
No	104	54.5		87	45.5	191	47.2			0.488 (0.324–0.735)	0.001*
**Had the knowledge of using the system of reporting**	**(*n* = 152)**			**(*n* = 62)**		**(*n* = 214)**					
Yes^b^	59		67.0	29	33.0	88	41.1	1.152	0.283	–	–
No	93		73.8	33	26.2	126	58.9			1.385(0.763–2.514)	0.284

*χ*^2^ chi-square test, *FE* Fisher exact,

*OR* odds ratio, *CI* confidence interval,

*LL* lower limit, *UL* upper limit

^*^ Statistically significant at *p* ≤ 0.05

^a^ More than one answer

^b^ Reference group

Accordingly, a multiple logistic regression analysis was conducted (Table 3). The work schedule (evening shifts) (*p* < 0.001), number of colleagues in the same place of work (6–10) (*p*: 0.003), and the presence of a system for reporting violence (*p*: 0.003) were the significant predictors for WPV. Table 4 showed that the most prevalent type of violence that occurred against healthcare workers was the verbal type of violence (87.9%), mainly from the patient’s relatives (74.6%), and 10.5% was a combination of verbal and physical. In addition, 96.1% of the exposed HCWs reported no physical injury from the violent event, and 71.5% deemed the violent incident preventable.

**Table 3:** Multivariate logistic regression analysis for the predictors affecting suffering from violence in context with the COVID-19 pandemic among the studied healthcare workers in Egypt

	***p***	**OR (95% *CI*)**
**Age (years)**	0.103	0.591 (0.314–1.112)
**Gender**	0.369	1.242 (0.774–1.992)
**Work schedule#**		
Evening	< 0.001*	2.807 (1.793–4.393)
**Number of colleagues in the same place of work**		
None	0.745	1.183 (0.429–3.265)
1–5	0.473	0.829 (0.497–1.383)
6–10	0.003*	0.393 (0.213–0.723)
> 10®		
**There is a system for reporting violence**	0.003*	1.928 (1.243–2.989)

*OR* odd’s ratio,

*CI* confidence interval, *LL* lower limit, *UL* upper limit

^#^ All variables with *p* < 0.05 were included in the multivariate

^*^ Statistically significant at *p* ≤ 0.05

**Table 4:** Characteristics of violent events against the exposed Egyptian HCWs during the COVID-19 pandemic in our study (*n* = 256)

Q	Characteristics of violent events against exposed HCWs	No	%
	**Kind of violence**		
	Physical	4	1.6
	Verbal	225	87.9
	Both	27	10.5
	**The violent person**^**a**^		
	Patient	74	28.9
	Relative	191	74.6
	Colleague	1	0.4
	Administrative	9	3.5
	Supervisor	3	1.2
	Others	33	12.9
	**Any physical injury from the violent event**		
	No	246	96.1
	Yes	10	3.9
	**Was the violent incident preventable?**		
	No	73	28.5
	Yes	183	71.5

^a^ More than one answer

**Fig. 3:**
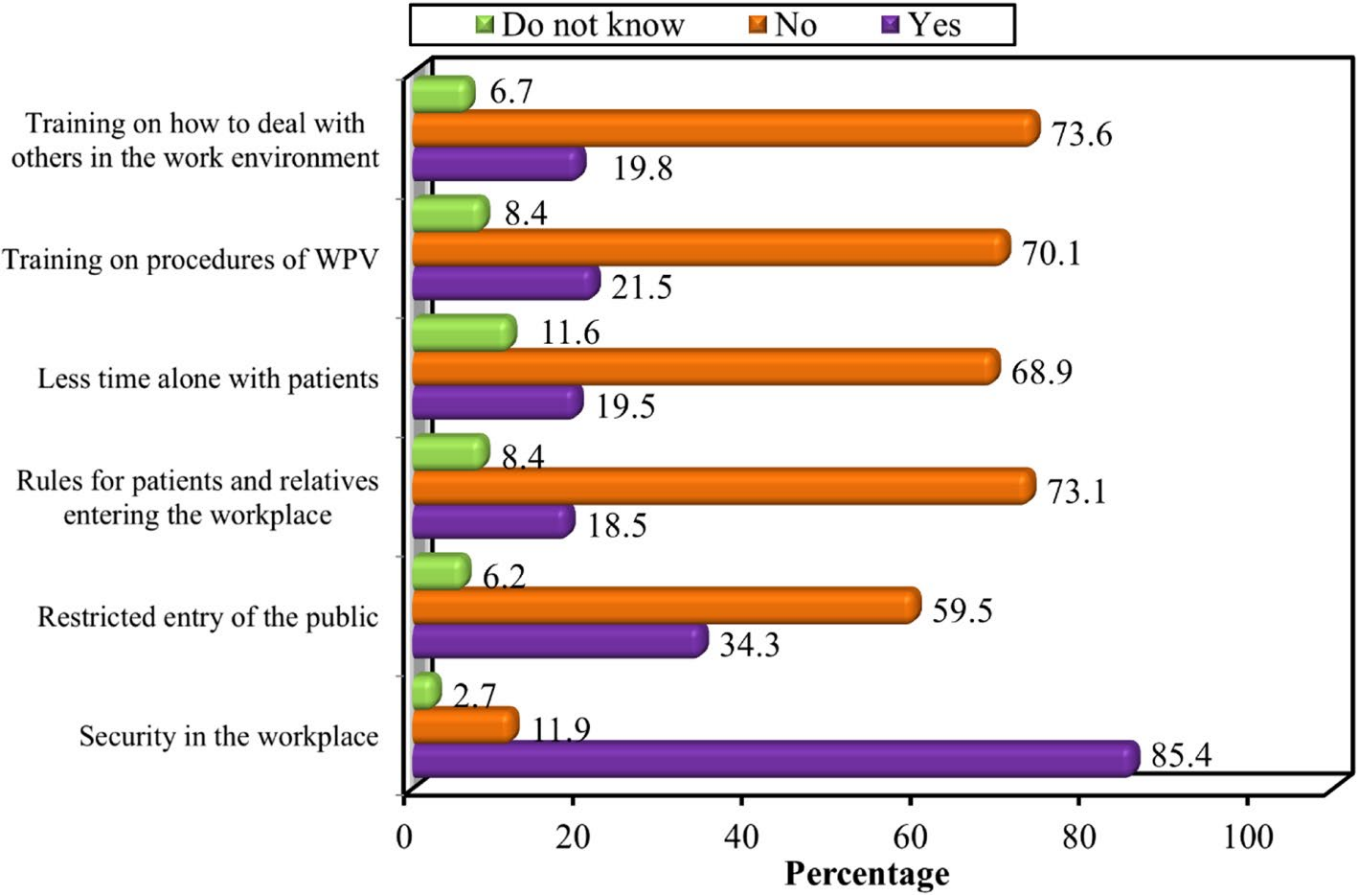
Opinion of the studied healthcare workers about workplace characteristics that can prevent exposure to violent events during COVID-19 pandemic

Figure 3 shows the opinions of HCWs regarding workplace characteristics that can prevent violent events. The majority (85.4%) of the studied HCWs believed that security in the workplace could diminish violent events. About 60% of the studied HCWs (59.5%) did not believe that restriction of public entry might affect the occurrence of violence at work. Only 18.5% of the studied HCWs thought that violence at work could be controlled if there were rules for patients and relatives entering the workplace. Spending less time alone with patients was reported as a reason to control violence at work by 19.5% of exposed HCWs, while nearly most of them (68.9%) did not know if such control would help in reducing violent events. About one-quarter of the studied HCWs (21.5%) reported that they need training on “working procedures of workplace violence” and on “how to deal with others in the work environment” (19.8%) as a measure to control violent incidents.

Table 5 elucidated the reasons behind the non-reporting of the violent events by the exposed HCWs, where 81.9% considered reporting non-beneficial and 12.1% did not know to whom they shall report. clarified the opinions of the studied HCWs regarding the causes of workplace violence events against HCWs. Almost a quarter of HCWs (25.9%) considered lack of capabilities as a direct cause of violence, 22.7% viewed that lack of adequate security had a role, 19.5% blamed the absence of deterrent law, 10.5% and 9.1% voted for ignorance and bad manners and unawareness, respectively, and 6.7% blamed the media for distorting the “doctor’s image.”

**Table 5:** Reasons for non-reporting exposure to violent events and the most important factors that cause incidents of violence during COVID-19 pandemic among the studied participants

	**No**	**%**
**Reasons for non-reporting among the exposed participants (*n* = 232)**^**a**^		
Feel ashamed	3	0.7
Considered not beneficial	190	81.9
I do not know who reported it	28	12.1
Others	18	7.8
**The most important factors that cause incidents of violence against the health team in your workplace (*n* = 405)**		
Unawareness	37	9.1
There is no deterrent law	79	19.5
Lack of capabilities	105	25.9
Ignorance and bad manners	43	10.6
Lack of respect for the medical team	14	3.5
The media and the distortion of the image of doctors	27	6.7
Absence of an effective security presence	92	22.7
Others	8	2.0

^a^ More than one answer

**Fig. 4:**
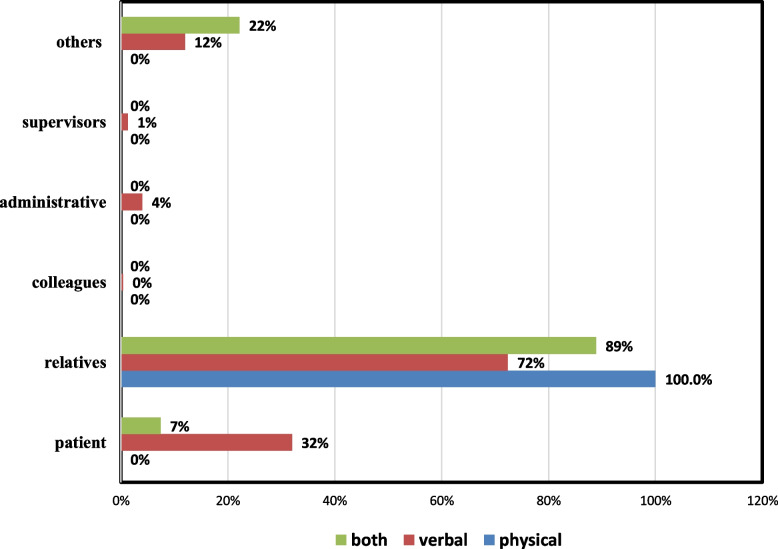
The frequency of the type of violence against HCWs according to the perpetrator of the violent event

Figure 4 clarified the frequency of type of violent event according to violent person. All relatives caused physical violence, 89% of them caused both type of violence, and nearly 70% of them caused verbal violence, while 32% of patient caused verbal violence. A total of 4% of administrative staff caused verbal violence.

## Discussion

Many studies showed that healthcare professionals are more likely to be victims of violence than workers in other professions, and the disturbing normalization of this problem exacerbates the rise in occurrences and personal implications for HCWs (Bitencourt et al., 2021). The situation differs during the COVID-19 pandemic, which has infected over 150 million individuals worldwide and resulted in more than three million deaths worldwide, even though the epidemic significantly influenced everyone’s mental health. Due to extended working hours, insufficient personal protective equipment (PPE), and the severe danger of contamination, frontline HCWs were the most exposed to its emotional impact. Although most communities worldwide recognized the critical role of HCWs during the epidemic, there was evidence that violence against them increased during the COVID-19 pandemic (Bhatti, Rauf, Aziz, Martins, & Khan, 2021; Bitencourt et al., 2021).

This is one of the first studies in Egypt to examine frequency and determinants of violence towards HCWs using a varied sample of different categories of HCWs in a number of Egyptian governorates during the COVID-19 epidemic.

This study showed that about two-thirds of HCWs (63.2%) reported being exposed to workplace violence during the pandemic which is considered high, comparable to that previously documented study before the pandemic in an emergency department in Egypt, and stated that workplace violence was reported by 59.7% of HCWs. Also, higher than a previously reported in a study conducted among Jordanian nurses in different departments at three hospitals in Amman stated that prevalence of verbal and physical abuse was 37.1% and 18.3%, respectively (Ahmed, 2012). Additionally, the estimated WPV was higher than previously reported in studies conducted in Saudi Arabia (45.6%) and in Turkey (44.7%) (Pinar et al., 2017). Approximately, near same prevalence was reported in the context of the pandemic in Nepal (64.9%) (Sahiran, Minhat, & Saliluddin, 2021) and in Jordan (65.5%) (Ghareeb, El-Shafei, Eladl, 2021).

Conversely, a higher prevalence was reported in China (83.3%) (Sun et al., 2017) and Saudi Arabia (67.4%) (Algwaiz & Alghanim, 2012), and a study was conducted at the Al-Zahraa University Hospital, which illustrated that 66.3% of HCWs had been exposed to violence (SA & WR, 2021). This difference in prevalence of violence between current study and previous studies may be due to the dissimilarity in the definition of violence, the duration of reporting of violence, the methodology used, and the culture of the study population. There is underestimation of the true prevalence of violence in our study because this study was conducted online which will miss HCWs who do not have enough time to Internet access during the pandemic. Another factor could be underreporting where the majority (90.6%) of HCWs exposed to violent incidents declared non-reporting. Although our study included different categories of HCWs from different Egyptian governorate, the sample is not representative of all HCWs in Egypt because the study is based on purposive sampling technique.

Verbal violence is usually a preliminary phase that may either extend to a physical one or be controlled. This may explain our findings regarding types of violence, where 87.9% of total HCWs had been exposed to verbal violence, 10.5% reported exposure to both verbal and physical violence, and only 1.6% reported exposure to physical violence. This agreed with other studies conducted in Egypt, in Mansoura University Hospitals (Abou-ElWafa, El-Gilany, Abd-El-Raouf, Abd-Elmouty, & El-Sayed Hassan El-Sayed, 2015), and in Tanta Emergency Hospital (Kabbash & El-Sallamy, 2019), in which verbal violence had the highest predominance. Similar findings were reported in India (Mishra et al., 2018) and China (Shi et al., 2017), where verbal abuse was as high as 75.9% and 64.9%, respectively. Also, in South Korea, verbal abuse was the highest (63.8%) and then physical violence (22.3%) (Park et al., 2015). Conversely, the prevalence of physical violence was the uppermost (35.6%) in the Palestinian hospitals. This higher prevalence may be explained by the stress created by war and the political conflicts in this area (Hamdan & Hamra, 2015).

Violence was significantly linked with younger age (< 40 years), which was 83.7%; this is in agreement with the results found by Gacki-Smith et al. (2009). They found that less work experience and younger age are more associated with violence; it might be due to the respect of the public for older healthcare workers (Gacki-Smith et al., 2009)*.*Violence was most prevalent against female HCWs (64.7%). This finding agreed with the study conducted in Iraq, where younger age groups dominated, with 44% being under age 30 and 37.2% between 30 and less than 40 years, especially in female HCWs (61.4%) (Lafta et al., 2021). This could be attributed to female HCWs being deemed weak and incapable of defending themselves by the offending persons.

Accordingly, multiple logistic regression analysis was conducted to detect the predictors of WPV exposure. The work schedule (evening shifts), number of colleagues in the same place of work (6–10), and the presence of a system for reporting violence were the significant factors for WPV (*p* < 0.05). As regards the increase in violent incidents in the evening shifts more than in the daytime shifts, these findings agreed with (Bilisli & Hizay, 2016), who declared that the evening or night shifts were the times when more than half of violent incidents occurred and explained that by defective security measures and less experience of junior staff in dealing with violent situations at night and in the evening shifts. However, this disagreed with (Kabbash & El-Sallamy, 2019), who declared that the physical and verbal violence happened equally in all shifts.

Furthermore, the current study showed that patient relatives were the most frequent perpetrators (74.6%), followed by the patients themselves (28.9%). These results agreed with the findings of previous studies in Egypt (Abdellah & Salama, 2017), India (Mishra et al., 2018), and Ethiopia (Tiruneh et al., 2016). This could be attributed to the presence of patients’ relatives during patients’ management, their excessive stress from fear of losing patients’ lives, not obeying the rules regarding the visiting time, and the presence of a weak security system.

Concerning the preventive measures against violence, the HCWs enrolled in our study regarded that strengthening security measures, training on the procedures of WPV, setting rules for patients and their relatives, restricting public access, and decreasing the time alone with the patient were the supreme repeated preventive measures (85.4%, 21.5%, 18.5%, 34.3%, and 19.5%, respectively). Similar suggested measures were reported by (Koukia, Mangoulia, Gonis, & Katostaras, 2013) and (Kumar et al., 2016). In Turkey, employee security units and legislation have been established to prevent violence against HCWs (Kumar et al., 2016).

A noteworthy finding in our study is that the majority (90.6%) of HCWs exposed to violent incidents declared non-reporting, either because they viewed reporting as non-beneficial (81.9%) or they did not know the mechanism of reporting (12.1%). This could be explained by the predominance of verbal abuse in our study (87.9%), where HCWs might skip reporting them because of increasing workload during the pandemic or appreciating the mental status of patients and their relatives, or, unfortunately, due to acceptance of such insults as “regular” due to their extensive widespread occurrence. This was consistent with the findings of a research carried out in Saudi Arabia, which claimed that the reasons for not reporting violence were linked to insufficient reporting processes, distrust of the reporting system, and a lack of confidence in the violence prevention system’s efficiency (Al-Shamlan et al., 2017). Towhari and Bugis (2020) found similarly that most occurrences were not reported, despite the existence of a formal reporting system, due to a deficiency of system privacy and the assumptions that WPV was part of one’s job responsibilities.

Almost half of the HCWs considered lack of workplace logistics a prime cause of violence, 25.9% lack of capabilities, and 22.7% lack of adequate security. In addition, 19.5% blamed the absence of a deterrent law, 10.5% and 9.1% voted for ignorance and bad manners and unawareness, respectively, and 6.7% blamed the media for distorting the “doctor’s image.” This agreed somehow with Towhari and Bugis (2020); they denoted the patient’s health condition, lack of staff, high workload, and the lack of security personnel as the major violence predictors. This agrees with Shastri (Shastri, 2019), who stated that the media had played a negative role by projecting a negative picture of healthcare malpractice. According to Towhari and Bugis (2020), the Saudi Ministry of Health’s initiative and penalties for reducing WPV have resulted in a considerable decrease in the prevalence of violence and have satisfied healthcare employees.

## Conclusions

During a health crisis, HCWs are the most vital treasure; thus, their safety and well-being should be a top priority. The frequency of violence against HCWs in the context of the COVID-19 pandemic is high. The most prevailing type of violence among HCWs was verbal violence. Patient relatives were the most frequent perpetrators. Being less than 40 years old, female, physician, and working in night shift increase the risk of exposure to violence. The majority of the exposed HCWs reported no physical injury from the violent event. A quarter of HCWs considered lack of capabilities as an important cause of violence. HCWs thought that strengthening the security in workplace could be an important factor to prevent exposure to violence. The majority of HCWs exposed to violent incidents declared non-reporting, either because they viewed reporting as non-beneficial or they did not know the mechanism of reporting. At both a national and international level, the issue of healthcare violence must be identified and addressed. To reduce violence and safeguard the safety of the medical profession, the government, health policymakers, media organizations, and community engagement groups must collaborate for healthcare workers’ safety.

### Limitations

There are certain limitations to our research. First, it is based on self-reported data, which can be biased. Second, the participants were asked if they had been exposed to violence in the previous 3 months, which could lead to recall bias. Finally, the study took into account the COVID-19 epidemic. As a result, the findings cannot be applied to HCWs in the usual situations.

## Data Availability

Raw data were generated at the SPSS sheet. Derived data supporting the findings of this study are available upon request.

## Abbreviations

HCWs: Healthcare workers
SPSS: Statistical Package for Social Science
WPV: Workplace violence
WHO: World Health Organization
